# Disproportionality analyses of acoltremon: a real-world safety assessment based on the the FDA adverse event reporting system database

**DOI:** 10.3389/fmed.2026.1906740

**Published:** 2026-08-06

**Authors:** Xiao Hu, Cong Yu, Yaping Huang

**Affiliations:** 1 Department of Pharmacy, Fujian Provincial Hospital, Fuzhou University Affiliated Provincial Hospital, Fuzhou, Fujian, China; 2 Department of Neurology, Fujian Provincial Hospital, Fuzhou University Affiliated Provincial Hospital, Fuzhou, Fujian, China

**Keywords:** acoltremon, drug safety, dry eye disease, FAERS, pharmacovigilance study

## Abstract

**Introduction:**

Acoltremon is a first-in-class transient receptor potential melastatin 8 agonist approved for the treatment of the signs and symptoms of dry eye disease. Given its increasing clinical use following recent approval, post-marketing evaluation of its adverse event (AE) profile is warranted. This study aims to offer insights into the characteristics of acoltremon association with AEs.

**Methods:**

Disproportionality analysis, including the reporting odds ratio, proportional reporting ratio, information component, and empirical Bayesian geometric mean, were employed in our study. AE signals are determined by using these algorithms. The database for analysis, sourced from the FDA Adverse Event Reporting System, covers the period from the second quarter of 2025 to the first quarter of 2026.

**Results:**

A total of 2,667 AEs associated with acoltremon were identified, with 45 positive signal preferred terms in our study. The three most frequently reported AEs during acoltremon treatment were eye irritation (*n* = 1,420), eye pain (*n* = 475) and ocular hyperaemia (*n* = 226), and the top three AE signals based on reporting odds ratio (ROR) values were eye irritation (380.62, 95% confidence interval (CI): 355.07–408.02), eye pain (129.42, 95% CI: 116.94–143.23) and abnormal sensation in eye (122.70, 95% CI: 78.77–191.14). Drug hypersensitivity (*n* = 45, ROR 4.15) was also identified as a disproportionality signal in this study. Notably, rhinorrhoea (*n* = 35, ROR 5.78), nasal discomfort (*n* = 5, ROR 12.37), ear pruritus (*n* = 4, ROR 8.09), ophthalmic herpes simplex (*n* = 3, ROR 57.73), respiratory tract infection viral (*n* = 3, ROR 8.12) were newly detected disproportionality signals identified. Comparative analysis with other dry eye therapies showed that several ocular AEs were also observed with comparator treatments, whereas some newly detected signals, including rhinorrhoea, nasal discomfort, ear pruritus and ophthalmic herpes simplex were not detected among the comparator drugs. Sensitivity analysis excluding reports with concomitant medications showed that the major disproportionality findings remained generally consistent, although several signals were no longer detected, including ear pruritus, ophthalmic herpes simplex, and respiratory tract infection viral.

**Discussion:**

This study identified potential safety signals associated with acoltremon in the FAERS database. These findings should be interpreted cautiously and warrant further pharmacovigilance and clinical investigation.

## 1 Introduction

Dry eye disease (DED) is a common and multifactorial disease of the ocular surface characterized by a loss of homeostasis of the tear film, leading to inflammation and damage to the corneal epithelial surface and conjunctiva (1). The pathogenesis of DED involves dysfunction of one or more ocular structures responsible for maintaining a stable tear film (2). The key etiological factors include tear film instability, hyperosmolarity, ocular surface inflammation and damage, as well as neurosensory abnormalities (3). Patients with DED typically experience ocular irritation symptoms of dryness, grittiness, foreign body sensation, burning, stinging, light sensitivity, and ocular pain, as well as vision-related symptoms such as blurred and/or fluctuating vision. These symptoms substantially impair patients' visual function, diminish physical and mental quality of life, reduce work productivity, and compromise activities of daily living (4–6). DED has emerged as a major global public health concern, affecting hundreds of millions of people worldwide (7).

As a chronic and frequently progressive disease, DED often requires long-term treatment (8). Current treatment strategies primarily include lubricants (artificial tears, gels, ointments, and autologous serum), corticosteroids, immunosuppressants (cyclosporine, tacrolimus, and lifitegrast), and a cholinergic agonist (a nasal spray formulation of varenicline) (9, 10). Despite the broad range of available therapies, conventional treatments remain limited by their modest efficacy, unpleasant adverse events (AEs), and slow onset of action (11–13).

Acoltremon is a first-in-class transient receptor potential melastatin 8 (TRPM8) agonist approved by the US Food and Drug Administration on May 28, 2025, for the treatment of the signs and symptoms of DED (14). TRPM8 ion channels, which are expressed on cold thermosensory neurons innervating the cornea and eyelid, serve as key regulators of basal tear production, and their dysfunction has been linked to the pathophysiology of DED (15). By activating TRPM8 signaling pathways, acoltremon increases tear production and demonstrates notable clinical benefits, including a rapid onset of action and significant improvement in both the signs and symptoms of DED (14). Acoltremon offers a novel and effective treatment option for patients with DED, with the potential to transform the current therapeutic landscape for DED.

The common AE associated with acoltremon was instillation site pain, predominantly characterized by mild burning and stinging. Conjunctival hemorrhage, visual acuity reduced, vision blurred, vitreous detachment have also been observed during the clinical trail of acoltremon (14). However, as a first-in-class drug that has only recently been approved, acoltremon is expected to see increasingly widespread clinical use. Real-world evidence regarding its long-term safety profile remains limited. Therefore, close monitoring in real-world clinical settings is urgently needed to further characterize its AE profile beyond clinical trials.

The FDA Adverse Event Reporting System (FAERS) serves as a large-scale repository for post-marketing AEs, and provides vast data for pharmacovigilance signal detection. Leveraging this database, we conducted a comprehensive pharmacovigilance analysis to characterize the early post-marketing safety profile of acoltremon in real-world clinical practice. By analyzing disproportionality signals, severe outcomes, and time-to-onset evaluations, this study aims to identify potential safety signals that may not have been fully recognized during pre-marketing clinical development. These findings will provide timely real-world evidence to guide clinical monitoring, support early detection of unexpected AEs, and promote safer use of acoltremon in routine ophthalmic practice.

## 2 Methods

### 2.1 Study design and data source

Disproportionality analysis undertaken within the confines of this study was structured as a case/non-case investigation, aiming to quantify the association between acoltremon and AEs. The proportion of target AEs occurring with acoltremon (designated as cases) in contrast to all other pharmaceutical agents (designated as non-cases) was calculated. A significant signal emerged when acoltremon showed an increased likelihood of inducing a specific AE compared with all other drugs collectively.

The data used for this analysis were obtained from the following web address: https://fis.fda.gov/extensions/FPD-QDE-FAERS/FPD-QDE-FAERS.html. To incorporate the latest case reports, data from the second quarter of 2025 to the first quarter of 2026, as recorded in the FAERS database, were extracted.

### 2.2 Data extraction

Following FDA guidelines, a deduplication process was implemented to ensure the integrity and uniqueness of the dataset (16). Cases linked to acoltremon were pinpointed by employing the terms “ACOLTREMON” and “TRYPTYR” as search criteria and selecting “Primary suspected” (PS). All AEs submitted to FAERS adhered to the standardized categorization of Medical Dictionary for Regulatory Activities 26.0 (MedDRA 26.0), which encompasses a five-tier hierarchy, comprising system organ class (SOC), high-level group term, high-level term, preferred term (PT), and lowest-level term (17).

**Figure 1:**
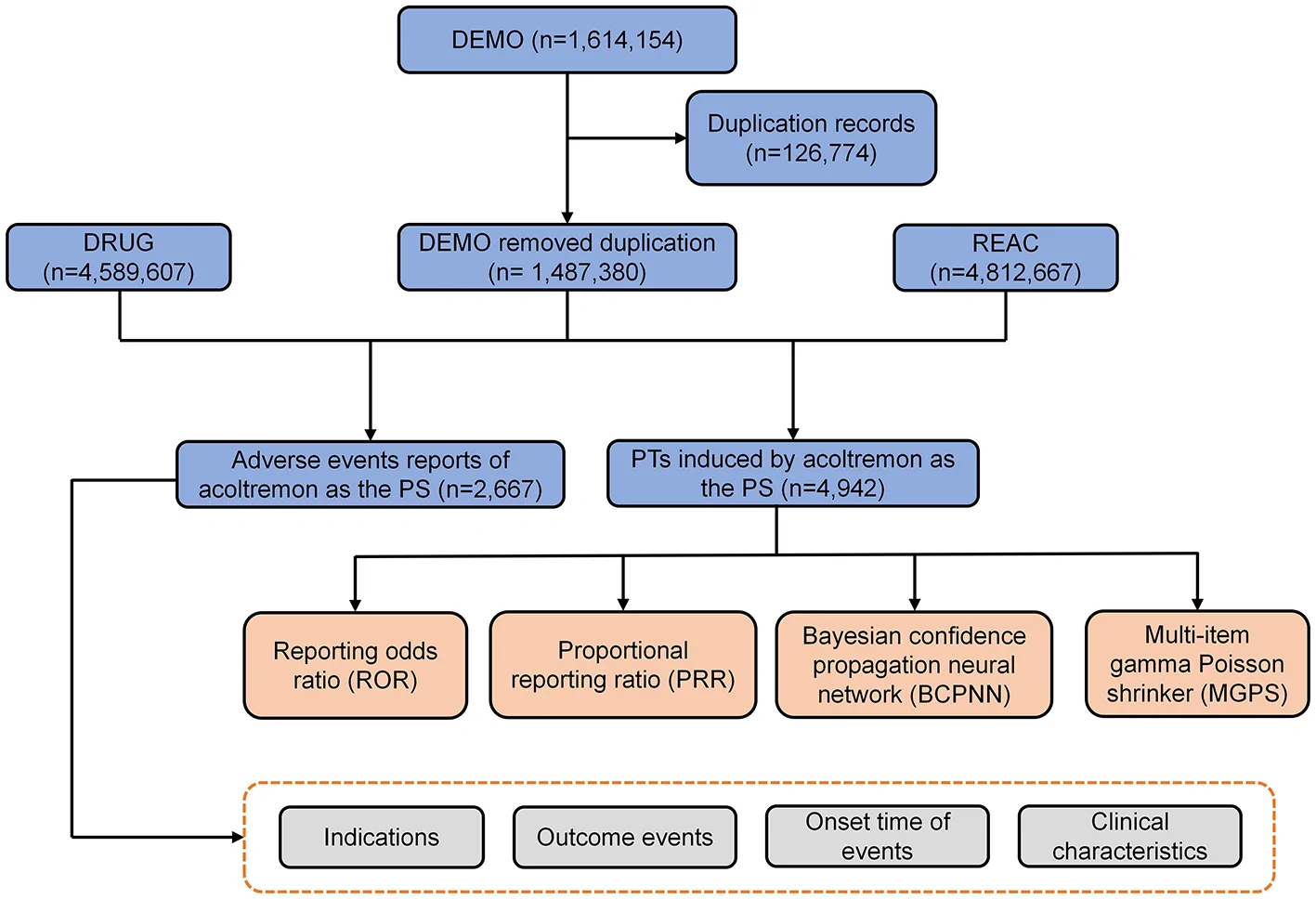
The flow diagram of selecting acoltremon-related AEs from FAERS database. AE, adverse event; FAERS, FDA adverse event reporting system.

A series of descriptive analyses were conducted to provide detailed information on the clinical features in reports associated with acoltremon. Where data permitted, detailed information was determined and presented, covering patient characteristics (such as age and weight), manifestation of severe outcomes, geographic distribution of reports, and reporter identities. This analytical process concluded with the construction of a visually informative flowchart (Figure 1).

### 2.3 Data analysis

In pharmacovigilance, a safety signal refers to information arising from one or multiple sources that suggests a new potentially causal association, or a new aspect of a known association, between a medicinal product and an AE, and that warrants further evaluation (18). Importantly, signal detection through disproportionality analysis does not establish causality but serves as a hypothesis-generating approach for identifying potential drug–event associations.

Signal identification relied on the simultaneous use of four distinct algorithms: reporting odds ratio (ROR), proportional reporting ratio (PRR), information component (IC), and empirical Bayesian geometric mean (EBGM). In the present study, an AE was deemed a signal only when it satisfied the predefined criteria across all four algorithms (19). The specific criteria for each algorithm were as follows:

1. ROR was used to assess the strength of the association between a specific drug and a given AE; a positive signal was defined when the lower bound of the 95% confidence interval of the ROR (ROR 05) exceeded 1.
2. PRR compared the proportion of a specific AE reported for the target drug with that for all other drugs in the database; a signal was considered present when PRR ≥ 2, the chi-square statistic ≥ 4, and the number of reports for the event was at least 3.
3. IC, a Bayesian-based signal detection metric, was calculated, with a statistically significant signal defined as a lower 95% credibility interval bound (IC025) greater than 0.
4. EBGM was employed to obtain shrinkage estimates and reduce small-sample bias; an abnormal reporting signal was identified when the lower bound of the EBGM credibility interval (EBGM05) exceeded 2.

### 2.4 Comparative analysis with other dry eye therapies

To partially address potential confounding by indication, a comparative analysis was performed using two other drugs indicated for DED, lifitegrast and perfluorohexyloctane, as comparator treatments. These drugs were selected because they share the same therapeutic indication and route of administration with acoltremon. Selected PTs were compared between acoltremon and comparator therapies, including the three most frequently reported AEs, the top three AE signals, and newly detected disproportionality signals.

### 2.5 Sensitivity analysis

To evaluate the potential influence of concomitant medications on the detected safety signals, a sensitivity analysis was performed by excluding FAERS reports in which concomitant drugs were reported. The disproportionality analysis was then repeated using the same predefined criteria for signal detection as in the primary analysis. The identified signals were compared between the primary analysis and the sensitivity analysis to assess the robustness of the findings.

### 2.6 Statistical analysis

For categorical variables, frequencies and corresponding percentages are provided. Non-normally distributed data are presented as median values with their interquartile range (IQR).

All data processing and statistical analyses were performed using Microsoft Excel 2019, R (version 4.4.2) and Python 3.10 (Python Software Foundation). R packages including dplyr, ggplot2, and epiR were used for data cleaning, signal calculation, and visualization.

## 3 Results

### 3.1 Descriptive analysis

After excluding duplicate reports, a total of 2,667 AEs associated with acoltremon were identified. The clinical characteristics of patients who experienced acoltremon-associated AEs were summarized in Table 1. Among all AE reports with available data, a higher proportion involved females, accounting for 87.81%, compared to males (12.19%). Regarding age distribution, acoltremon-related AEs were most frequently reported in patients older than 65 years (69.73%), followed by those aged 18 to 65 years (30.15%) and those younger than 18 years (0.12%). The median age of patients with acoltremon-associated AE reports was 71 years [interquartile range (IQR) 64–78 years]. In terms of reported indications, dry eye was the more frequently documented condition (90.51%). The majority of AE reports originated from the United States (99.78%), with minor contributions from Colombia and China. A low proportion of serious AEs was documented for acoltremon, accounting for 1.69%. Healthcare professionals submitted the majority of acoltremon-related AE reported (74.14%).

**Table 1:** Clinical characteristics of AE reports with acoltremon from the FAERS database.

Characteristics	Acoltremon-induced AE reports (*n* = 2,667)		
Number of events	Available number, *n*	Case number, *n*	Case proportion
Gender, *n* (%)	2,526	–	94.71%
Female	–	2,218	87.81%
Male	–	308	12.19%
Age (years), *n* (%)	1,652	–	61.94%
< 18	–	2	0.12%
18 ≤ and ≤ 65	–	498	30.15%
> 65	–	1,152	69.73%
Median (IQR)	–	71 (64–78)	–
Weight (Kg), *n* (%)	4	–	0.15%
< 80	–	2	50.00%
80 ≤ and ≤ 100	–	2	50.00%
> 100	–	0	0.00%
Median (IQR)	–	73 (59–85)	–
Reported countries, *n* (%)	2,667	–	100.00%
United States	–	2,661	99.78%
Colombia	–	4	0.15%
China	–	2	0.07%
Indications, *n* (%)	411	–	15.41%
Dry eye	–	372	90.51%
Combination drugs, *n* (%)	1,524	–	57.14%
Levothyroxine	–	343	22.51%
Atorvastatin	–	227	14.90%
Rosuvastatin	–	205	13.45%
Outcomes, *n* (%)	2,663	–	99.85%
Non-serious outcome	–	2,618	98.31%
Serious outcome^a^	–	45	1.69%
Death	–	0	0.00%
Life-threatening	–	1	2.22%
Hospitalization	–	14	31.11%
Disability	–	0	0.00%
Other serious outcomes	–	35	77.78%
Time-to-onset (days)	128	–	4.80%
Median (IQR)	–	0 (0–1)	–
Reporters, *n* (%)	2,664	–	99.89%
Health professional	–	1,975	74.14%
Consumer	–	689	25.86%
Reporting year^b^, *n* (%)	2,667	–	100.00%
2025	–	1,302	48.82%
2026	–	1,365	51.18%

^a^Total serious outcomes may exceed the total number of reported cases because some cases list more than one serious outcomes.

^b^The second quarter of 2025 to the first quarter of 2026.

AE, adverse event; FAERS, FDA adverse event reporting system; IQR, interquartile range.

### 3.2 Disproportionality analysis

Signal reports of acoltremon at the SOC level are presented in Table 2. The occurrence of AEs related to acoltremon was distributed across 22 different organ systems. The significant SOC related to acoltremon that met the criteria of all algorithms was eye disorders (*n* = 1,982, ROR 58.17).

**Table 2:** Signal strength of SOCs of acoltremonb in FAERS database.

SOC	Number	ROR (95% two-sided CI)	PRR (χ^2^)	IC (IC025)	EBGM (EBGM05)
**Eye disorders** ^a^	**1,982**	**58.17 (54.41–62.20)**	**26.19 (47,278.71)**	**4.66 (4.36)**	**25.25 (24.33)**
General disorders and administration site conditions	737	1.32 (1.22–1.43)	1.25 (45.57)	0.33 (0.30)	1.25 (1.18)
Immune system disorders	67	1.21 (0.95–1.54)	1.21 (2.39)	0.27 (0.21)	1.20 (0.97)
Ear and labyrinth disorders	18	1.04 (0.65–1.65)	1.04 (0.02)	0.05 (0.03)	1.01 (0.67)
Nervous system disorders	191	0.75 (0.65–0.87)	0.76 (15.16)	−0.39 (−0.45)	0.76 (0.68)
Respiratory, thoracic and mediastinal disorders	97	0.62 (0.51–0.76)	0.63 (21.95)	−0.66 (−0.81)	0.63 (0.53)
Skin and subcutaneous tissue disorders	100	0.48 (0.39–0.58)	0.49 (55.11)	−1.02 (−1.24)	0.49 (0.41)
Social circumstances	9	0.36 (0.19–0.69)	0.36 (10.25)	−1.47 (−2.83)	0.34 (0.19)
Injury, poisoning and procedural complications	149	0.26 (0.22–0.31)	0.29 (295.01)	−1.77 (−2.08)	0.29 (0.26)
Infections and infestations	46	0.22 (0.16–0.29)	0.23 (127.82)	−2.13 (−2.85)	0.23 (0.17)
Cardiac disorders	16	0.21 (0.13–0.34)	0.21 (48.37)	−2.25 (−3.67)	0.20 (0.13)
Gastrointestinal disorders	48	0.18 (0.13–0.24)	0.19 (181.22)	−2.41 (−3.20)	0.19 (0.14)
Psychiatric disorders	16	0.11 (0.07–0.18)	0.11 (115.57)	−3.14 (−5.13)	0.11 (0.07)
Surgical and medical procedures	6	0.07 (0.03–0.16)	0.07 (70.69)	−3.75 (−8.35)	0.07 (0.03)
Hepatobiliary disorders	3	0.07 (0.02–0.22)	0.07 (37.01)	−3.82 (−11.84)	0.06 (0.02)
Musculoskeletal and connective tissue disorders	10	0.06 (0.03–0.12)	0.07 (138.54)	−3.92 (−7.30)	0.06 (0.04)
Vascular disorders	5	0.06 (0.02–0.14)	0.06 (75.16)	−4.05 (−9.74)	0.05 (0.03)
Investigations	10	0.05 (0.03–0.10)	0.06 (166.84)	−4.15 (−7.72)	0.05 (0.03)
Reproductive system and breast disorders	1	0.04 (0.01–0.27)	0.04 (24.09)	−4.69 (−33.32)	0.02 (0.00)
Metabolism and nutrition disorders	3	0.04 (0.01–0.11)	0.04 (78.16)	−4.77 (−14.81)	0.03 (0.01)
Renal and urinary disorders	2	0.03 (0.01–0.13)	0.03 (59.33)	−4.96 (−19.83)	0.02 (0.01)
Blood and lymphatic system disorders	1	0.01 (0.00–0.10)	0.01 (69.61)	−6.13 (−43.54)	0.01 (0.00)

The bold data indicated the four distinct algorithms's results of positive signal.

^a^Positive signal.

CI, confidence interval; EBGM, empirical Bayesian geometric mean; FAERS, FDA adverse event reporting system; IC, information component; PRR, proportional reporting ratio; ROR, reporting odds ratio; SOC, system organ class; χ^2^, chi-squared.

In total, 45 positive signal PTs were observed in our study (Table 3). The three most frequently reported AEs during acoltremon treatment were eye irritation (*n* = 1,420), eye pain (*n* = 475) and ocular hyperaemia (*n* = 226), and the top three AE signals based on ROR values were eye irritation (380.62, 95% confidence interval (CI): 355.07–408.02), eye pain (129.42, 95% CI: 116.94–143.23) and abnormal sensation in eye (122.70, 95% CI: 78.77–191.14). Drug hypersensitivity (*n* = 45, ROR 4.15) was also identified as a disproportionality signal in this study. Notably, rhinorrhoea (*n* = 35, ROR 5.78), nasal discomfort (*n* = 5, ROR 12.37), ear pruritus (*n* = 4, ROR 8.09), ophthalmic herpes simplex (*n* = 3, ROR 57.73), respiratory tract infection viral (*n* = 3, ROR 8.12) were newly detected disproportionality signals identified in the present study.

**Table 3:** Signal strength of PTs related to acoltremonb in FAERS database (*n* ≥ 3).

PTs	Number	ROR (95% CI)	PRR (χ^2^)	IC (IC025)	EBGM (EBGM05)
Eye irritation	1,420	380.62 (355.07–408.02)	271.55 (277442.53)	7.62 (7.11)	196.75 (188.31)
Eye pain	475	129.42 (116.94–143.23)	117.07 (46985.88)	6.65 (6.01)	100.57 (93.20)
Ocular hyperaemia	226	50.93 (44.37–58.45)	48.64 (9881.06)	5.51 (4.80)	45.49 (40.72)
Vision blurred	216	23.65 (20.59–27.16)	22.66 (4343.23)	4.46 (3.88)	21.94 (19.59)
Lacrimation increased	120	47.10 (39.08–56.77)	45.98 (4962.67)	5.43 (4.51)	43.07 (36.97)
Ocular discomfort	113	91.92 (75.43–112.01)	89.84 (8816.83)	6.32 (5.19)	79.53 (67.93)
Eye pruritus	98	24.53 (20.02–30.06)	24.06 (2097.13)	4.54 (3.71)	23.19 (19.58)
Eye swelling	50	19.91 (15.01–26.41)	19.72 (865.12)	4.26 (3.22)	19.03 (14.97)
Visual impairment	45	4.39 (3.27–5.90)	4.36 (116.13)	2.12 (1.58)	4.29 (3.33)
Drug hypersensitivity	45	4.15 (3.09–5.57)	4.12 (105.92)	2.04 (1.52)	4.06 (3.15)
Rhinorrhoea	35	5.78 (4.14–8.08)	5.75 (136.42)	2.51 (1.80)	5.63 (4.22)
Photophobia	27	20.22 (13.78–29.68)	20.12 (477.16)	4.29 (2.92)	19.23 (13.83)
Eye discharge	26	27.71 (18.71–41.04)	27.57 (641.10)	4.73 (3.20)	26.07 (18.63)
Foreign body sensation in eyes	25	59.59 (39.59–89.70)	59.30 (1322.75)	5.78 (3.84)	53.72 (38.11)
Burning sensation	23	5.30 (3.51–8.00)	5.28 (79.30)	2.39 (1.59)	5.14 (3.59)
Abnormal sensation in eye	23	122.70 (78.77–191.14)	122.13 (2358.57)	6.71 (4.30)	102.13 (71.35)
Swelling of eyelid	23	24.39 (16.08–36.99)	24.28 (496.59)	4.56 (3.00)	23.00 (16.07)
Periorbital swelling	22	20.85 (13.63–31.89)	20.76 (402.18)	4.34 (2.84)	19.74 (13.67)
Erythema of eyelid	20	44.93 (28.57–70.66)	44.75 (804.95)	5.40 (3.43)	41.11 (27.94)
Eye inflammation	19	18.72 (11.86–29.54)	18.65 (309.29)	4.19 (2.65)	17.72 (11.92)
Eyelid margin crusting	14	35.17 (20.55–60.20)	35.08 (441.74)	5.07 (2.96)	32.29 (20.24)
Eyelid irritation	14	59.11 (34.25–102.03)	58.95 (736.51)	5.77 (3.34)	52.58 (32.96)
Vitreous floaters	11	11.97 (6.59–21.74)	11.95 (108.57)	3.56 (1.96)	11.24 (6.60)
Conjunctivitis	10	5.40 (2.89–10.06)	5.39 (35.47)	2.42 (1.30)	5.09 (2.90)
Eye disorder	10	4.04 (2.17–7.53)	4.03 (22.70)	2.01 (1.08)	3.82 (2.18)
Sneezing	8	4.24 (2.11–8.50)	4.23 (19.64)	2.07 (1.03)	3.95 (2.09)
Blepharitis	8	20.66 (10.22–41.74)	20.63 (145.19)	4.33 (2.14)	18.83 (9.99)
Eye infection	8	7.56 (3.76–15.18)	7.55 (44.99)	2.90 (1.45)	7.02 (3.72)
Photopsia	7	17.02 (8.04–36.04)	17.00 (102.96)	4.06 (1.92)	15.45 (7.80)
Asthenopia	7	16.74 (7.91–35.43)	16.71 (101.06)	4.03 (1.90)	15.20 (7.67)
Eye hemorrhage	7	9.07 (4.30–19.12)	9.06 (49.54)	3.16 (1.50)	8.32 (4.20)
Eyelids pruritus	7	20.11 (9.48–42.65)	20.08 (123.46)	4.29 (2.02)	18.18 (9.18)
Intraocular pressure increased	6	8.16 (3.65–18.25)	8.15 (37.20)	3.01 (1.35)	7.40 (3.51)
Madarosis	6	24.15 (10.70–54.51)	24.12 (128.62)	4.55 (2.01)	21.45 (10.18)
Nasal discomfort	5	12.37 (5.11–29.95)	12.35 (51.29)	3.60 (1.49)	10.97 (4.79)
Facial pain	5	6.45 (2.67–15.57)	6.45 (22.80)	2.68 (1.11)	5.77 (2.52)
Hordeolum	5	8.09 (3.35–19.55)	8.09 (30.70)	3.00 (1.24)	7.22 (3.15)
Blepharospasm	5	5.15 (2.14–12.43)	5.15 (16.60)	2.36 (0.98)	4.62 (2.02)
Ear pruritus	4	8.09 (3.02–21.69)	8.09 (24.56)	3.00 (1.12)	7.03 (2.74)
Eye injury	4	12.22 (4.55–32.86)	12.22 (40.50)	3.59 (1.33)	10.56 (4.11)
Ocular icterus	3	13.18 (4.21–41.32)	13.18 (33.15)	3.70 (1.18)	10.87 (3.53)
Hypoaesthesia eye	3	73.65 (22.43–241.87)	73.61 (194.73)	6.06 (1.85)	56.03 (18.21)
Ophthalmic herpes simplex	3	57.73 (17.79–187.29)	57.69 (154.60)	5.74 (1.77)	44.83 (14.57)
Respiratory tract infection viral	3	8.12 (2.60–25.35)	8.12 (18.51)	3.01 (0.96)	6.74 (2.19)
Eyelid pain	3	25.43 (8.04–80.46)	25.41 (67.93)	4.62 (1.46)	20.61 (6.70)

CI, confidence interval; EBGM, empirical Bayesian geometric mean; FAERS, the FDA adverse event reporting system; IC, information component; PRR, proportional reporting ratio; PT, preferred terms; ROR, reporting odds ratio; χ^2^, chi-squared.

### 3.3 Comparison with other dry eye therapies

To explore the potential influence of confounding by indication, we compared selected PT-level disproportionality signals associated with acoltremon with those reported for lifitegrast and perfluorohexyloctane, two drugs used for DED (Supplementary Table 1). Eye irritation, eye pain, ocular hyperaemia, and abnormal sensation in eye were also reported as signals with comparator therapies. These signals showed higher reporting disproportionality with acoltremon. Rhinorrhoea, nasal discomfort, ear pruritus and ophthalmic herpes simplex were not observed as signals among the comparator drugs. Furthermore, respiratory tract infection viral remained newly detected disproportionality signals in this comparison.

### 3.4 Sensitivity analysis

Among the 2,667 reports included in the primary analysis, 1,524 reports (57.15%) involved concomitant medications, whereas 1,143 reports (42.85%) reported acoltremon without concomitant drugs. After exclusion of concomitant medication reports, the major disproportionality findings remained generally consistent (Supplementary Table 2). Eye irritation and eye pain remained the two most frequently reported AEs and the top two AE signals. However, ocular hyperaemia and abnormal sensation in eye identified in the primary analysis were replaced by vision blurred and ocular discomfort as the three most frequently reported AEs and the top three AE signals. Rhinorrhoea and nasal discomfort remained detectable after excluding concomitant medication reports, whereas ear pruritus, ophthalmic herpes simplex, and respiratory tract infection viral were no longer detected.

### 3.5 Time-to-onset of acoltremonb-related AEs

**Figure 2:**
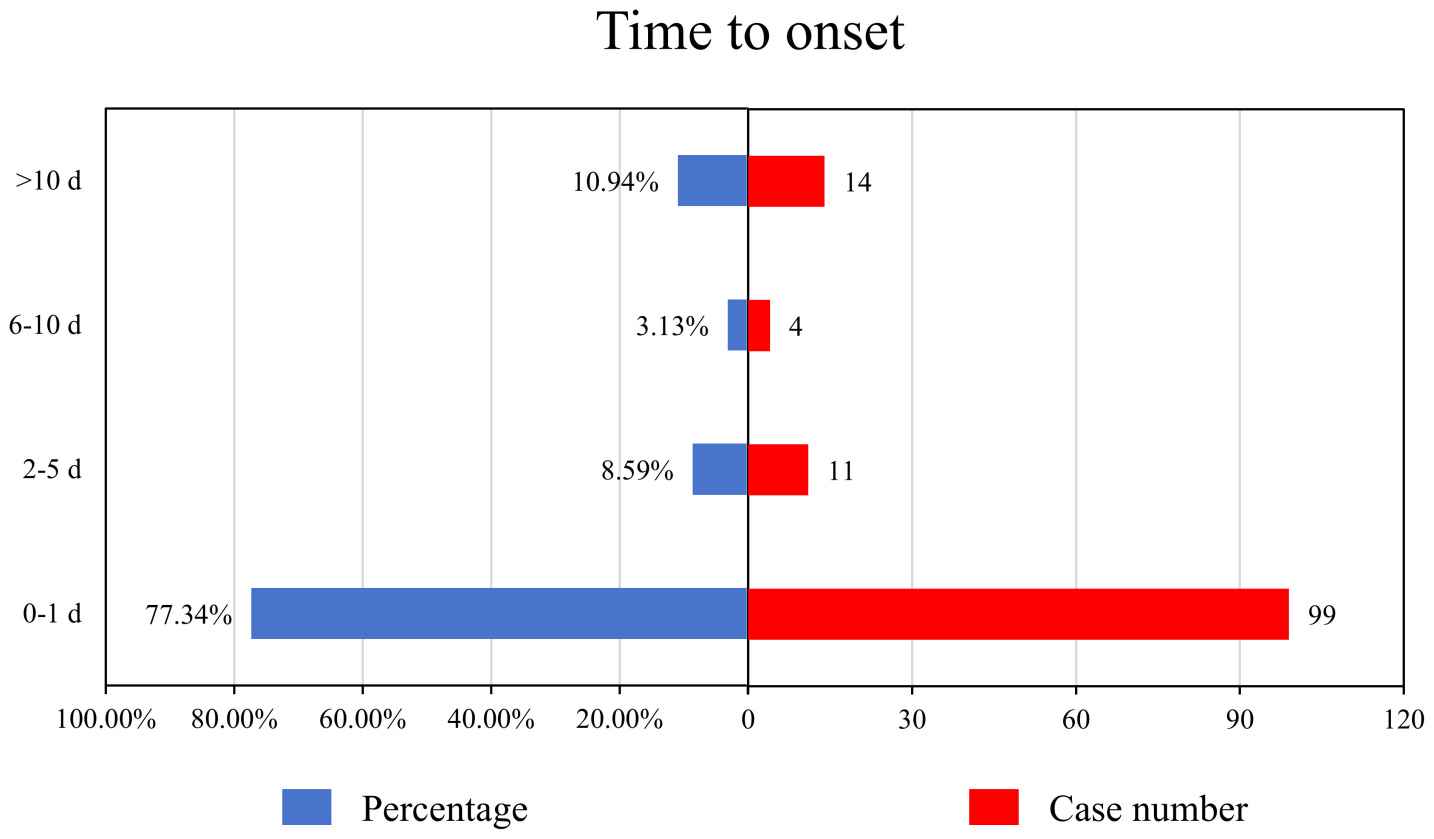
Time-to-onset of acoltremonb-related AEs. AE, adverse event.

From April 2025 to March 2026, approximately 4.80% of patients had reported onset times, revealing a median time-to-onset of 0 days (IQR 0–1 days). The majority of acoltremon-associated AEs occurred within 0–1 days after initial treatment (*n* = 99, 77.34%), followed by 2–5 days (*n* = 11, 8.59%), 6–10 days (*n* = 4, 3.13%), and >10 days (*n* = 14, 10.94%). Detailed information on the time-to-onset of acoltremon-related AEs is shown in Figure 2.

## 4 Discussion

It is widely recognized that AEs and drug safety are critical considerations in clinical therapeutics, as they may compromise patient adherence and, in severe cases, necessitate treatment discontinuation. A comprehensive understanding of AEs is therefore indispensable not only for their prevention and mitigation but also for their timely identification and management in routine clinical practice. Nevertheless, given the recent approval of acoltremon, post-marketing safety experience remains limited, highlighting the importance of continued pharmacovigilance to further characterize its AE profile in real-world settings. In the present study, we employed a pharmacovigilance approach to characterize disproportionality signals associated with acoltremon-related AEs in real-world settings.

Among the detected signals, ocular symptoms including eye irritation, eye pain, and ocular hyperaemia were among the most frequently reported events and were biologically plausible considering the known pharmacological activity of TRPM8 activation. In contrast, newly identified signals not previously described in the product label, particularly infection-related signals, require further evaluation to clarify their clinical significance.

Previous studies have suggested that acoltremon possesses a favorable safety profile, with the vast majority of treatment-emergent AEs reported to be mild in severity (14). Consistent with these findings, our analysis demonstrated that acoltremon was documented in a lower proportion of serious AEs than non-serious AEs (1.69% vs. 98.31%). These findings are consistent with the generally favorable tolerability profile observed in clinical trials, however, they should be interpreted cautiously because FAERS-based analyses cannot establish causality or evaluate long-term safety outcomes.

Our study identified eye irritation, eye pain and ocular hyperaemia as the three most frequently reported AEs associated with acoltremon. Consistently, the top three AE signals based on ROR values were eye irritation, eye pain and abnormal sensation in eye. These findings are biologically plausible considering the known pharmacological activity of acoltremon as a TRPM8 agonist. TRPM8 receptors are expressed at the nerve endings of two types of corneal cold thermosensory neurons: low-threshold (LT) and high-threshold (HT) neurons. LT neurons play a pivotal role in regulating basal tear production (20, 21), whereas HT neurons are implicated in the transmission of a cooling sensation accompanied by a mild irritative component (20–22). When applied to the corneal surface, acoltremon stimulates substantial tear production, which we hypothesize to be driven largely by LT cold thermosensory neurons (23). In contrast, the stimulation of HT cold thermosensory neurons may lead to the transient transmission of a cold sensation that may be interpreted as burning or stinging in some patients (24, 25). This mechanistic basis plausibly explains the high reporting frequency and strong signal strength of eye irritation and eye pain observed in our study. Abnormal sensation and ocular hyperaemia may represent a secondary response to local sensory irritation. Overall, our findings from real-world data corroborate the tolerability profile of acoltremon established in clinical trials, where instillation site pain (including mild burning and stinging) was the most common AE.

Drug hypersensitivity (*n* = 45, ROR 4.15) was also identified as a disproportionality signal in this study, which was not listed in the drug labeling. However, It can be found in some non-regulatory drug information sites (for example, https://www.webmd.com/drugs/tryptyr-acoltremon). Patients should be informed about possible hypersensitivity symptoms, and suspected cases should be appropriately evaluated in clinical practice. Further post-marketing studies are warranted to clarify its clinical significance.

Rhinorrhoea (*n* = 35, ROR 5.78), nasal discomfort (*n* = 5, ROR 12.37) and ear pruritus (*n* = 4, ROR 8.09) were also found in our study, which were not documented in the drug label. From an anatomical perspective, these findings can be reasonably explained by the interconnected drainage pathways between the eye, nasal cavity, and middle ear. The nasolacrimal duct provides a direct conduit from the ocular surface to the inferior meatus of the nasal cavity, allowing topically applied ophthalmic solution to flow into the nasal cavity after instillation (26). Once in the nasal cavity, the solution may reach the nasopharynx and, in some cases, the torus tubarius of the Eustachian tube, which connects the nasopharynx to the middle ear (27). Therefore, the observed nasal and ear symptoms are likely attributable to the mechanical spread of the drug via natural anatomical pathways and subsequent local mucosal contact. Clinicians should be aware of these potential drug-related symptoms, inform patients about their possible occurrence, and closely monitor for them during treatment. In most cases, nasal or ear discomfort following acoltremon instillation is mild, although persistent or unusual symptoms warrant further clinical consideration.

Notably, ophthalmic herpes simplex (*n* = 3, ROR 57.73) and respiratory tract infection viral (*n* = 3, ROR 8.12) emerged as newly detected disproportionality signals in the present study. Experimental evidence suggests that TRPM8 signaling may participate in antiviral responses. An animal model study demonstrated that loss of TRPM8 function exacerbates herpes simplex keratitis, resulting in increased corneal viral load and enhanced infiltration of CD11b^+^Ly6G^+^ inflammatory cells, whereas TRPM8 activation with menthol alleviated the infection, suggesting a potential protective role of TRPM8 against herpes simplex virus type 1 in the cornea (28). Furthermore, TRPM8 has been reported to influence T-cell activation and proliferation (29), and to counteract transient receptor potential cation channel subfamily V member 1-mediated pro-viral effects (30). Rhinovirus infection has also been shown to upregulate TRPM8 expression in human neuronal cells, indicating a potential interaction between TRPM8 signaling and respiratory viral infection processes (31). However, the mechanisms underlying the infection-related signals observed in this study remain uncertain. Although pharmacological modulation of TRPM8 signaling represents a possible biological hypothesis, the observed associations cannot be directly attributed to acoltremon based on FAERS data alone. Alternative explanations, including underlying ocular diseases, concomitant medications, patient susceptibility, and administration-related factors, should also be considered. In particular, improper handling or administration of ophthalmic products may result in contamination and increase the risk of ocular infections, which cannot be distinguished from drug-related effects in spontaneous reporting databases. Therefore, these findings should be interpreted as potential safety signals rather than confirmed drug-induced AEs. Given these infection-related signals, clinicians may consider maintaining awareness of potential infectious complications during acoltremon treatment, particularly in patients with a history of recurrent herpetic keratitis or immunocompromised status. Appropriate clinical evaluation and monitoring for signs of ocular or respiratory infection may be considered when clinically indicated. Although pharmacological mechanisms may provide biological plausibility for some observed signals, these interpretations remain hypothesis-generating and should not be considered evidence of causality. Further experimental and clinical studies are required to determine whether these signals represent true safety concerns and to validate the potential involvement of TRPM8 modulation in infection susceptibility.

Because acoltremon is used for DED, potential confounding by indication should be considered when interpreting AEs. The comparison with other drugs used for DED demonstrated that several ocular AEs were also observed with comparator treatments, indicating that some events may reflect disease-related factors or characteristics of topical ophthalmic formulations. Nevertheless, these events showed stronger disproportionality signals with acoltremon may be related to its TRPM8 agonistic activity. In addition, rhinorrhoea, nasal discomfort, ear pruritus and ophthalmic herpes simplex, which were newly detected signals in our study, were not observed as signals among the comparator drugs, suggesting that these events may warrant further evaluation in relation to acoltremon treatment. While respiratory tract infection viral remained newly detected disproportionality signals in this comparison. These findings support cautious interpretation of the results and highlight the need for further clinical evaluation.

To further assess the potential influence of concomitant medications on the detected signals, a sensitivity analysis was conducted by excluding reports with concomitant drug exposure. The major findings remained generally consistent, with eye irritation and eye pain continuing to represent the two most frequently reported AEs and the top two AE signal. These results suggest that the primary ocular signals associated with acoltremon were unlikely to be solely attributable to concomitant medication exposure. However, changes in the ranking of some PTs and attenuation of several newly detected signals after exclusion of concomitant medications highlight the potential impact of concomitant drugs on individual signal detection, particularly for events with limited numbers of reports. Therefore, these findings should be interpreted cautiously, and further clinical studies are required to clarify the independent contribution of acoltremon to these potential safety signals.

This study provides a comprehensive safety overview of acoltremon in clinical practice using large-scale, real-world data. However, our study inevitably has some limitations. First, FAERS is a spontaneous reporting system and is subject to reporting bias, under-reporting, stimulated reporting, duplicate reporting, and incomplete clinical information. Therefore, disproportionality analyses can detect potential safety signals but cannot establish causal relationships, estimate incidence rates, or determine the absolute risk of AEs. Second, the FAERS database does not provide access to cases' narratives, which greatly limits the analysis of the imputability of cases. Third, confounding factors, including patient comorbidities, concomitant medications, and potential drug-drug interactions, may have influenced the observed AE profiles. Although sensitivity analysis excluding concomitant medication reports demonstrated that major signals were generally robust, the potential contribution of concomitant drugs cannot be completely excluded due to the inherent limitations of spontaneous reporting systems.

## 5 Conclusion

Based on the FAERS database, this pharmacovigilance study provides a comprehensive assessment of the safety profile of acoltremon in real-world settings. The three most frequently reported AEs during acoltremon treatment were eye irritation, eye pain and ocular hyperaemia, and the top three AE signals based on ROR values were eye irritation, eye pain and abnormal sensation in eye. Drug hypersensitivity (*n* = 45, ROR 4.15) was also identified as a disproportionality signal in this study. Notably, rhinorrhoea (*n* = 35, ROR 5.78), nasal discomfort (*n* = 5, ROR 12.37), ear pruritus (*n* = 4, ROR 8.09), ophthalmic herpes simplex (*n* = 3, ROR 57.73), respiratory tract infection viral (*n* = 3, ROR 8.12) were newly detected disproportionality signals identified. Comparative analysis with other dry eye therapies showed that several ocular AEs were also observed with comparator treatments, whereas some newly detected signals, including rhinorrhoea, nasal discomfort, ear pruritus and ophthalmic herpes simplex were not detected among the comparator drugs. Sensitivity analysis excluding reports with concomitant medications showed that the major disproportionality findings remained generally consistent, although several signals were no longer detected, including ear pruritus, ophthalmic herpes simplex, and respiratory tract infection viral. This study identified potential safety signals associated with acoltremon in the FAERS database. These findings should be interpreted cautiously and warrant further pharmacovigilance and clinical investigation.

## Data Availability

The datasets presented in this study can be found in online repositories. The names of the repository/repositories and accession number(s) can be found in the article/ Supplementary material.
