# Supplementary material for: Disproportionality analyses of acoltremon: a real-world safety assessment based on the the FDA adverse event reporting system database
Source: Front Med (Lausanne). 2026 Aug 6;13:1906740. doi: 10.3389/fmed.2026.1906740 (PMC13489795; doi:10.3389/fmed.2026.1906740)
Supplement: Supplementary file 1 [file Supplementary_File_1.docx]

| **PT** | **acoltremon** | **other drugs** |
| --- | --- | --- |
|  | **n (ROR, 95%CI)** | **n (ROR, 95%CI)** |
| Eye irritation | 1420 (380.62, 355.07-408.02) | 152 (28.82, 24.45-33.98) |
| Eye pain | 475 (129.42, 116.94-143.23) | 80 (22.63, 18.09-28.32) |
| Ocular hyperaemia | 226 (50.93, 44.37-58.45) | 56 (14.86, 11.39-19.39） |
| Abnormal sensation in eye | 23 (122.70, 78.77-191.14) | 5 (29.98, 12.29-73.10) |
| rhinorrhoea | 35 (5.78, 4.14-8.08) | - |
| nasal discomfort | 5 (12.37, 5.11-29.95) | - |
| ear pruritus | 4 (8.09, 3.02-21.69) | - |
| ophthalmic herpes simplex | 3 (57.73, 17.79-187.29) | - |
| respiratory tract infection viral | 3 (8.12, 2.60-25.35) | 3 (10.39, 3.33-32.43) |

**Supplementary Table 1** Comparison of major AE signals associated with acoltremon and other drugs (lifitegrast and perfluorohexyloctane ) used for for dry eye disease.

“-” indicates that the PT was not meet the predefined signal detection criteria.

Abbreviations: n,number; PT, preferred terms; ROR, reporting odds ratio.

**Supplementary Table2** Comparison of the major disproportionality signals before and after excluding reports with concomitant medications.

| **Signal category** | **PT** | **Primary analysis(all reports)** | **Sensitivity analysis (excluding concomitant drugs)** |
| --- | --- | --- | --- |
|  |  | **n (ROR, 95%CI)** | **n (ROR, 95%CI)** |
| three most frequently reported AEs | Eye irritation | 1420 (380.62, 355.07-408.02) | 614 (356.31, 322.44-393.74) |
|  | Eye pain | 475 (129.42, 116.94-143.23) | 199 (126.20, 108.55-146.73) |
|  | Ocular hyperaemia | 226 (50.93, 44.37-58.45) | - |
|  | Vision blurred | - | 85 (23.13, 18.59-28.79) |
| top three AE signals | Eye irritation | 1420 (380.62, 355.07-408.02) | 614 (356.31, 322.44-393.74) |
|  | Eye pain | 475 (129.42, 116.94-143.23) | 199 (126.20, 108.55-146.73) |
|  | Abnormal sensation in eye | 23 (122.70, 78.77-191.14) | - |
|  | Ocular discomfort | - | 57 (111.29, 84.87-145.94) |
| Newly detected disproportionality signals | rhinorrhoea | 35 (5.78, 4.14-8.08) | 17 (7.10, 4.40-11.46) |
|  | nasal discomfort | 5 (12.37, 5.11-29.95) | 4 (25.00, 9.31-67.14) |
|  | ear pruritus | 4 (8.09, 3.02-21.69) | # |
|  | ophthalmic herpes simplex | 3 (57.73, 17.79-187.29) | # |
|  | respiratory tract infection viral | 3 (8.12, 2.60-25.35) | # |

“-” indicates that thePT was not ranked among the top three signals in the corresponding analysis, rather than absence of reporting or loss of signal

“#” indicates that the PT was not meet the predefined signal detection criteria after excluding reports with concomitant medications.

Abbreviations: n,number; PT, preferred terms; ROR, reporting odds ratio.
